# An Overview of AI-Assisted Design-on-Simulation Technology for Reliability Life Prediction of Advanced Packaging

**DOI:** 10.3390/ma14185342

**Published:** 2021-09-16

**Authors:** Sunil Kumar Panigrahy, Yi-Chieh Tseng, Bo-Ruei Lai, Kuo-Ning Chiang

**Affiliations:** Advanced Microsystem Packaging and Nano-Mechanics Research Laboratory, Department of Power Mechanical Engineering, National Tsing Hua University, Hsinchu 300, Taiwan; sunilpanigrahy.nit@gmail.com (S.K.P.); b6321963@yahoo.com.tw (Y.-C.T.); vincent9o134@gmail.com (B.-R.L.)

**Keywords:** FEM simulation, WLP, AI, machine learning, ANN, RNN, SVR, KRR, KNN, RF, regression model

## Abstract

Several design parameters affect the reliability of wafer-level type advanced packaging, such as upper and lower pad sizes, solder volume, buffer layer thickness, and chip thickness, etc. Conventionally, the accelerated thermal cycling test (ATCT) is used to evaluate the reliability life of electronic packaging; however, optimizing the design parameters through ATCT is time-consuming and expensive, reducing the number of experiments becomes a critical issue. In recent years, many researchers have adopted the finite-element-based design-on-simulation (DoS) technology for the reliability assessment of electronic packaging. DoS technology can effectively shorten the design cycle, reduce costs, and effectively optimize the packaging structure. However, the simulation analysis results are highly dependent on the individual researcher and are usually inconsistent between them. Artificial intelligence (AI) can help researchers avoid the shortcomings of the human factor. This study demonstrates AI-assisted DoS technology by combining artificial intelligence and simulation technologies to predict wafer level package (WLP) reliability. In order to ensure reliability prediction accuracy, the simulation procedure was validated by several experiments prior to creating a large AI training database. This research studies several machine learning models, including artificial neural network (ANN), recurrent neural network (RNN), support vector regression (SVR), kernel ridge regression (KRR), K-nearest neighbor (KNN), and random forest (RF). These models are evaluated in this study based on prediction accuracy and CPU time consumption.

## 1. Introduction

Electronics packaging plays an important role in the semiconductor industry. Currently, the mainstream electronic packaging structures include heterogeneous packaging, 3D packaging, system-in-packaging (SiP), fan-out (FO) packaging, and wafer-level packaging [1,2,3,4,5,6,7,8]. With the increasing complexity of packaging structures, manufacturing reliability test vehicles, and conducting ATCT experiments have become time-consuming and very expensive processes, the design-on-experiment (DoE) methodology for packaging design is becoming infeasible. As a result of the wide adoption of finite element analysis [9,10,11,12,13,14,15], accelerated thermal cycling tests are reduced significantly in the semiconductor industry, and package development time and cost are reduced as well. In a 3D WLP model, Liu [16] applied the Coffin–Manson life prediction empirical model to predict the reliability life of a solder joint within an accurate range. However, the results of finite element simulations are highly dependent on the mesh size, and there is no guideline to help researchers address this issue. Therefore, Chiang et al. [17] proposed the concept of “volume-weighted averaging” to determine the local strain, especially in critical areas. Tsou [18] successfully predicted packaging reliability through finite element simulation with a fixed mesh size in the critical area of the WLP structure. However, the results of simulation analysis are highly dependent on the individual researcher, and the results are usually inconsistent between simulations. In order to overcome this problem, the present work comparatively reviews an artificial intelligence (AI) approach in which electronic packaging design using a machine learning algorithm [19,20]. The use of machine learning for the analysis of electronic packaging reliability is the best way to obtain a reliable prediction result and meet the time-to-market demand. 

In recent years, AI theory has been widely used in various research domains. Machine learning involves the use of AI theory combined with big data to guide computers for training and learning; eventually, a simple AI model with input and output relationships will be developed to help researchers make design decisions [21,22,23,24]. Machine learning [25,26,27,28] can be applied for regression or classification models using either supervised or unsupervised learning. In this review, because the input datasets are labeled, the learning algorithm for predicting the reliability life is considered supervised. Several machine learning algorithms exist, such as artificial neural network (ANN), support vector regression (SVR), K-nearest neighbor (KNN), kernel ridge regression (KRR), recurrent neural network (RNN), random forest (RF), and convolutional neural network (CNN). 

The ANN is one of the most common machine learning methods. McCulloch and Pitts [29] proposed ANNs that mimic biological neural structures, using different numbers of hidden layers and neurons to construct different neural network structures. Denoeux [30] also explored data classification using a neural network algorithm. In this algorithm, the data are entered at the input layer and then calculated in the hidden layer. Once the calculation is completed, the result is shown in the output layer. RNN is a class of ANN algorithms in which connections between nodes form a directed network along a temporal sequence, making them more suitable for deep learning [31,32] when a large number of nonlinear datasets is available [33,34].

SVR, proposed by Cortes and Vapnik [35], is suitable for high-dimensional features, but it has not suited for a small amount of dataset. The concept of SVR is similar to the support vector machine (SVM), which is used to solve classification problems. SVM finds the best classifier by searching the hyperplane with the largest margin [36]. SVR is widely adopted in many fields, including biological, behavioral research, image analysis, and medical research [37,38,39]. Along with SVR, KRR is among the most popular kernel-based methods. Kernel-based methods are useful for nonlinear structural datasets [40]. KRR is simpler and faster to train with its closed-form solution, and it can outperform SVR [41]. Non-parametric calibration models eliminate the normality assumption and can represent almost any type of data, whether they are scattered or follow a certain trend. However, the models can exhibit only one type of prediction behavior, i.e., they cannot combine local and general prediction. Local calibration models, such as KNN interpolation, consider the surrounding neighborhood as input to obtain information about the output [42,43]. KNN is suitable for both classification and regression problems. The KNN classification output is decided by the nearest neighborhood, which depends on more number of nearest neighbors belongs to that class, whereas the regression output is decided by the average of the nearest neighbor value [44,45]. 

KNN and RF are more suitable for classification tasks; these two algorithms also show prominent performance for regression tasks. The RF algorithm was proposed in 2001 by Breiman [46]. An RF is formed by combining multiple classifications and regression trees (CART); it analyses [47,48] the data features and data distribution to generate multiple decision trees with different structures and finally summarizes the prediction results of all decision trees. 

The CNN is a machine learning algorithm for image recognition and image classification. In 2012, AlexNet, proposed by Krizhevsky et al. [49], achieved the highest accuracy in the Image Net competition; consequently, the CNN has become a focus in academia and has developed rapidly [50,51]. 

This study reviews the use of ANN, RNN, SVR, KRR, KNN, and RF for the reliability life cycles prediction of WLP. The aim is to learn and establish a regression model for the relationship between packaging geometries (input) and life cycle (output) results. Before the implementation of the above AI algorithms, an AI training database was generated using a finite element simulation combined with the Coffin–Manson empirical equation for WLP reliability life cycles prediction, and this standard simulation procedure was experimentally verified before the generation of the massive simulation database.

The rest of this paper is presented as follows: Section 2 implemented the finite element method for WLP. Section 3 discusses different types of machine learning models. Section 4 is the result and discussion for the machine learning models, and finally, we ended with some concluding remarks in Section 5.

## 2. Finite Element Method for WLP

**Figure 1:**
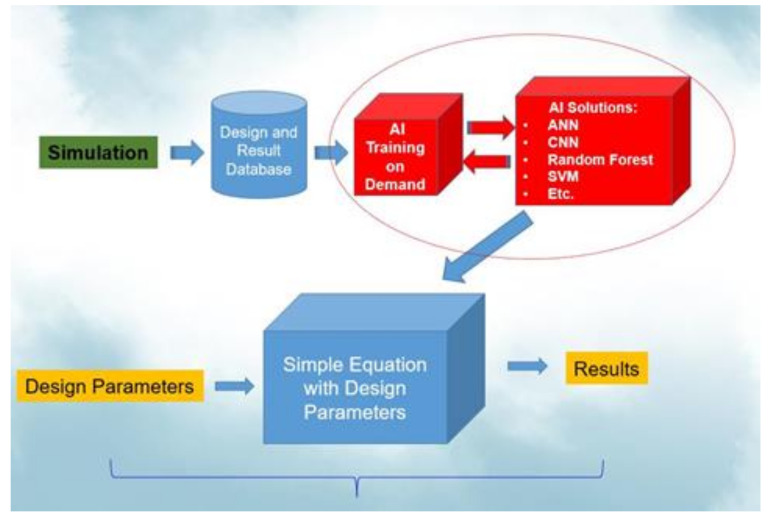
AI-assisted design-on-simulation procedure.

If the simulation consistently predicts the result of the experiment [52], then the simulation is an experiment; the experimental work can be replaced by a validated simulation procedure to create a large database for AI training and obtain a small and accurate AI model for reliability life cycles prediction. Once we obtain the final AI model for a new WLP structure, developers can simply input the WLP geometries, and then the life cycle can be obtained. Figure 1 illustrates this procedure. 

**Figure 2:**
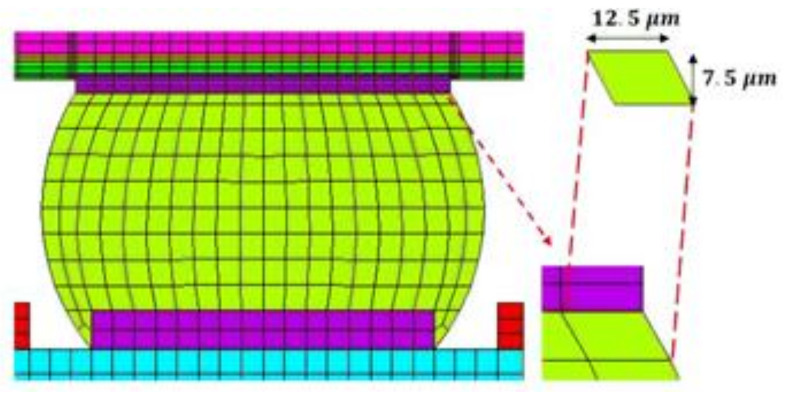
The critical mesh size.

Because a huge amount of data are required to build the AI training model, this work used a two-dimensional finite element method (FEM) model for simulation. Before the database is built, the simulation process must be reliable. This work validated the simulation results with five WLP test vehicles (Table 1 and Table 2). All of the sizes and specifications for different materials and the mean times to failure of the test vehicles are presented in the tables (Table 1 and Table 2) [53,54]. The simulation method mainly adopted a fixed mesh size at a critical location, determined through appropriate mechanics concepts, and an empirical equation was used to validate the reliability life cycles of all test vehicles. This work used the fixed mesh size of the solder joint at the maximum distance of the neutral point of the WLP to fix the modeling pattern and simulation procedure, as proposed by Tsou et al. [18]. As shown in Figure 2, the width and height of the fixed mesh size were 12.5 μm and 7.5 μm, respectively. The solder joint geometry was generated using Surface Evolver [55].

**Figure 3:**
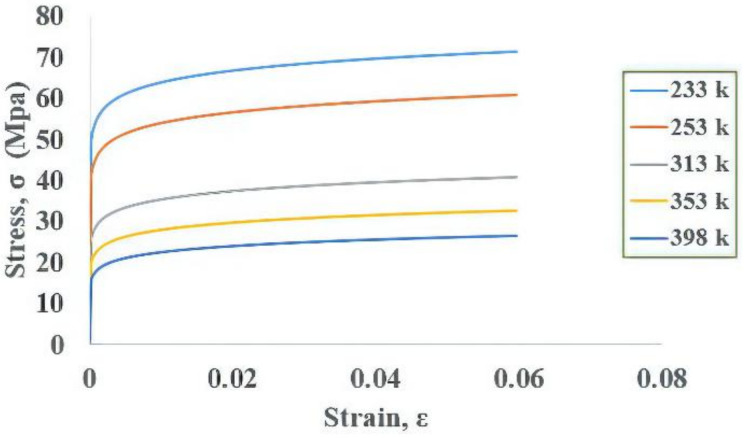
Stress–strain curve for SAC solder.

In the simulation process, the solder material was a nonlinear plastic material. Therefore, PLANE182, which has good convergence characteristics and can deal with large deformations, was used as the solder ball element. This work used PLANE42 for other components, which had linear material properties. Table 3 presents the list of individual material properties. The Young’s moduli at different temperatures of the solder joint are listed in Table 4. Figure 3 shows the stress–strain curve for an Sn–Ag–Cu (SAC)305 solder joint. The stress–strain curve [56], obtained by tensile testing and the Chabochee kinematic hardening model, was used to describe the tensile curves at different temperatures. Once the model is built, boundary conditions and external thermal loading are required for the WLP simulation.

**Figure 4:**
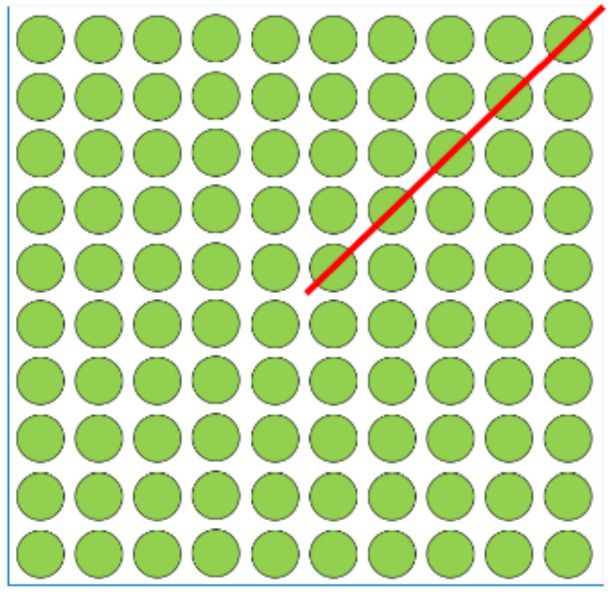
Symmetrical solder ball geometry.

**Figure 5:**
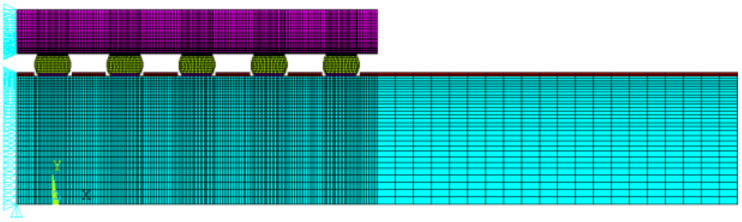
FEM model boundary condition.

Electronics packaging geometry is usually symmetrical; therefore, in this study, half of the 2D structure was modeled along the diagonal, as shown in Figure 4. The X-direction displacement on each node was fixed to zero owing to the Y-symmetry. To prevent rigid body motion, the node at the lowest point of the neutral axis, which is at the printed circuit board (PCB), has all degrees of freedom fixed. The complete finite element model and the boundary conditions are shown in Figure 5. The thermal loading condition used in this research was JEDEC JESD22-A104D condition G [57], and the temperature range was −40 °C to 125 °C. The ramp rate was fixed at 16.5 °C/min and the dwell time was 10 min. In a qualified design, its mean-cycle-to-failure (MTTF) should pass 1000 thermal cycles. After the simulation process is completed, the incremental equivalent plastic strain in the critical zone is substituted into the strain-based Coffin–Manson model [58] for reliable life cycle prediction. For a fixed temperature ramp rate, this method is as accurate as the energy-based empirical equation [59,60] but with much less CPU time.

The empirical formula for Coffin–Manson equivalent plastic strain model is shown in Equation (1):

(1) $$N_{f}=C{\left(\Delta \varepsilon_{eq}^{pl}\right)}^{-\eta }$$

where $N_{f}$ is mean cycle to failure, *C* and *η* are empirical constants, and $\Delta \varepsilon_{eq}^{pl}$ is the incremental equivalent plastic strain. For SAC solder joint, C and η are 0.235 and 1.75 [61,62].

Table 5 presents the predicted reliability life cycles of the WLP structure. The results show that the difference between the FEM-predicted life cycle and experiment result is within a small range. Therefore, experiments can be replaced by this validated FEM simulation to minimize the cost and time. Compared with the experiment approach, this validated FEM simulation procedure can provide large amounts of data within much less time and can be effectively used to generate a database for AI training. 

## 3. Machine Learning

Machine learning is an AI methodology that processes huge datasets to guide a computer for training, learning, and finally, building a simple regression model. In this study, several different machine algorithms were used, including ANN, RNN, SVR, KRR, KNN, and RF, implemented using the Python language. A supervised regression model, e.g., the WLP reliability life cycle prediction model, requires both input data (geometry parameters) and the corresponding output result (life cycles) for machine learning algorithms to build the final AI model of the WLP package. Once the regression model is established for a new WLP structure, the designer can simply input the WLP geometries of each component into the AI regression model to obtain the reliability life of this new WLP. This is a powerful and reliable technique for new packaging design.

### 3.1. Establishment of Dataset

**Figure 6:**
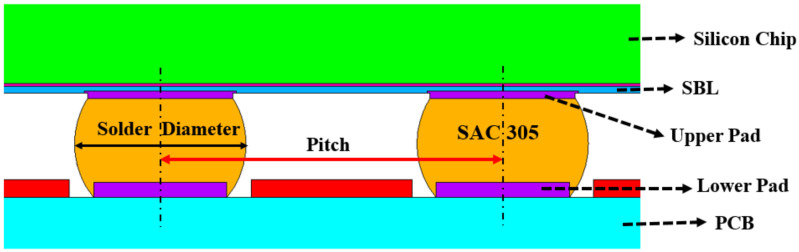
WLP geometry structure.

The WLP structure consists of several components, including the solder mask, solder ball, I/O pad, stress buffer layer, and silicon chip, etc. (Figure 6). For illustration purposes, the four most influential parameters, namely silicon chip thickness, stress buffer layer thickness, upper pad diameters, and lower pad diameters, were selected to build the AI model and predict the reliability life cycles of new WLP structures. These four design parameters were used to generate both training and testing datasets for AI machine learning algorithms. Table 6 and Table 7 show the generated training dataset obtained through FEM simulation. First, the number of training features generated in this research was 576 (Table 6), and 1296 (Table 7) datasets. For testing, 54 features were selected randomly from the interpolation of the above training dataset, which can help to build the AI training model. By increasing the training dataset, AI performance improves; however, the CPU/GPU time also increases.

### 3.2. ANN Model

**Figure 7:**
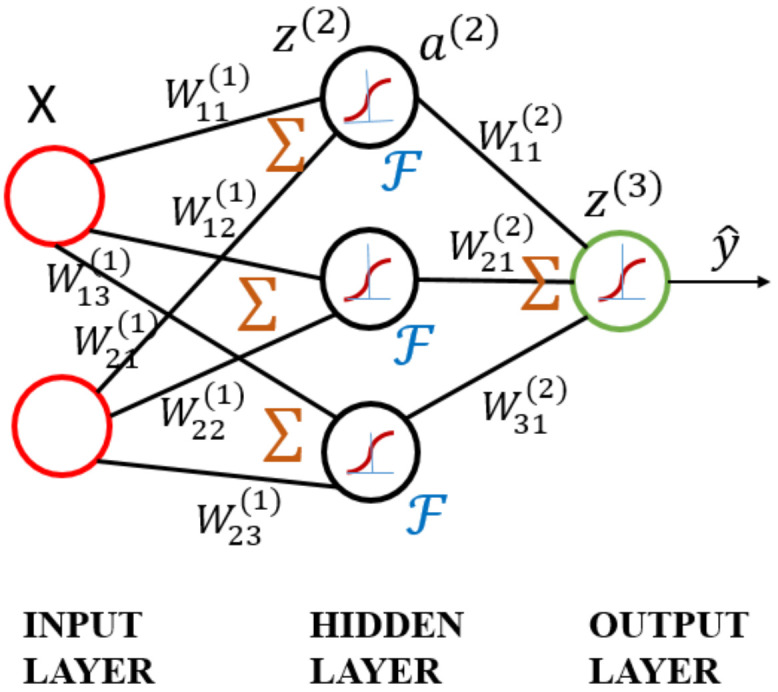
Schematic diagram of artificial neural network.

The ANN model is based on the concept of the brain’s self-learning ability, mimicking the human nervous system to process information. It is a multilayer neural network, as shown in Figure 7. The model consists of three layers: the input layer, where the data are provided; the hidden layer, where the input data are calculated; and the output layer, where the results are displayed [63]. As the numbers of neurons and hidden layers are increased, the ability to handle nonlinearity improves. However, these conditions may result in high computational complexity, overfitting, and poor predictive performance. 

In the above ANN model, $a_{i}^{l}$ is the ith activation element of the lth layer in the hidden layer. $b_{i}^{l}$ is bias, $a_{i}^{l}$ is equal to input value times weight $W_{ji}^{l}$ and add the bias in Equation (2):

(2) $$z_{i}^{l}=\sum_{i=1}^{n}w_{ji}^{l}a_{i}^{l}+b_{i}^{l}$$

**Figure 8:**
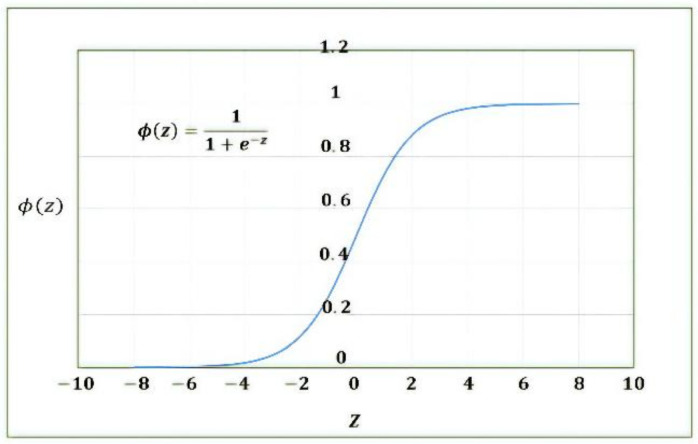
Sigmoid activation function.

From the calculation point of view, at first, the input layer data combined with bias and weight to obtain some value. The calculated input value, i.e., $a_{i}^{l}$, is substituted into activation function, e.g., Sigmoid, to be converted into a nonlinear form as an input of $a_{i}^{l+1}$ for the next layer.

(3) $$a_{i}^{l+1}=\phi (z_{i}^{l})$$

where the activation function is shown in Figure 8.

### 3.3. RNN Model

**Figure 9:**
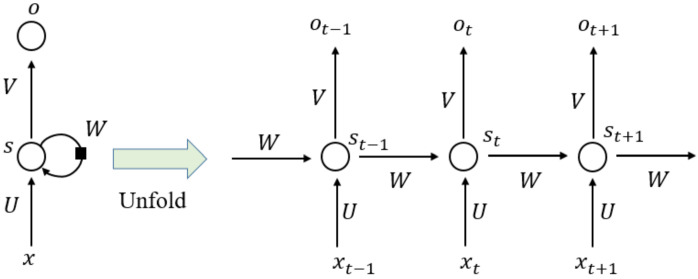
Schematic structure of recurrent neural network.

**Figure 10:**
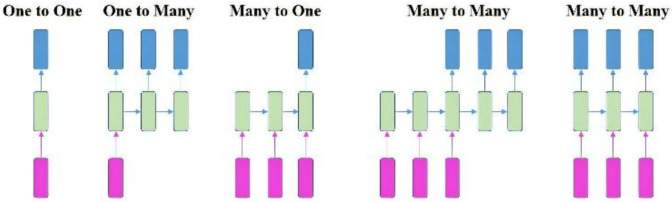
Different series model for RNN.

RNN is a type of neural network that can model “time-like”-series data, and it commonly adopts a nonlinear structure in deep learning. RNN [64,65] works on the principle that the output of a particular layer is fed back to the input layer to realize a time-dependent neural network and a dynamic model. Consequently, an ANN with nodes connected in a ring shape is obtained, as shown in the left half of Figure 9. The ring-shaped neural network is expanded along the “time” axis, as shown in the right half of Figure 9, where the “time” step t and the hidden state $s_{t}$ can be expressed as a function of the output from the previous ($s_{t-1}$) “time” steps and previous layers ($x_{t}$). U, V, and W denote the shared weights in RNN models during different “time” steps. Generally, the RNN series model can be divided into four types according to the number of inputs and outputs in given “time” steps; that is, one to one (O to O), one to many (O to M), many to one (M to O), and many to many (M to M). To synchronize the input features with the output results, RNN models can be subdivided into different series models, as shown in Figure 10 [66]. 

### 3.4. SVR Model

**Figure 11:**
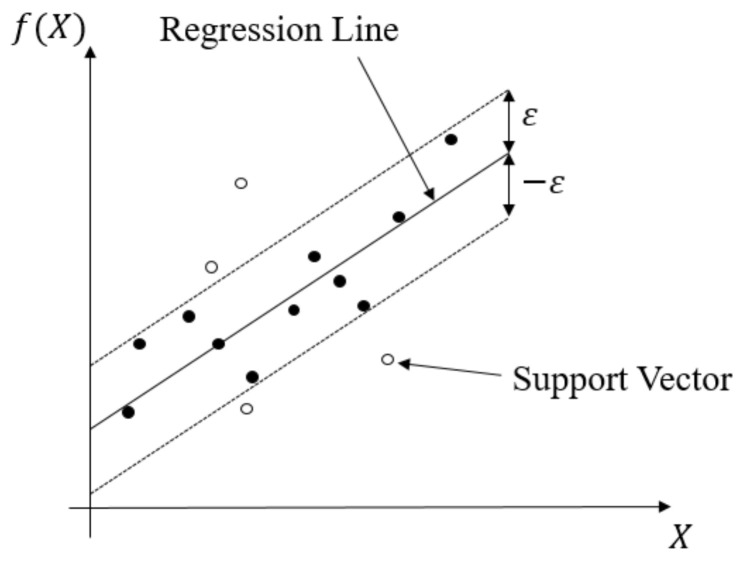
Schematic diagram of SVR.

This regression method evolved from the support vector machine algorithm. It transforms data to high-dimensional feature space and adapts the ε-insensitive loss function (Equation (4)) to perform the linear regression in feature space (Equation (5)). In this regression method, the norm value of w is also minimized to avoid the overfitting problem. In other words, $f\left(X,w\right)$, which is the function of the SVR model, will be as flat as possible. The SVR concept is illustrated in Figure 11. The data points outside the *ε*-insensitive zone are called support vectors, and two slack variables, $\xi_{i}$ and $\xi_{i}^{*}$, are used to record the loss of each support vector. Thus, the whole SVR problem can be seen as an optimization problem (Equation (6)).

(4) $$L(y,f(X))=\left\{\begin{array}{l} 0\\ \left|y-f(X)\right|-\varepsilon \; \end{array}\right.\left.\begin{array}{l} if\left|y-f(X)\right|\leq \varepsilon \;\\ otherwise \end{array}\right\}$$

(5) $$f(X)=\langle w,\phi (X)\rangle +b$$

(6) $$\begin{array}{l} \mathrm{minimize}\;\frac{1}{2}{\left\|w\right\|}^{2}+C\sum_{i=1}^{1}\xi_{i}+\xi_{i}^{*}\\ \mathrm{subject}\;\mathrm{to}\;\left\{\begin{array}{c} y_{i}-\langle w,\phi (X)\rangle -b\leq \varepsilon +\xi_{i}\\ \langle w,\phi (X)\rangle +b-y_{i}\leq \varepsilon +\xi_{i}^{*}\\ \xi_{i},\xi_{i}^{*}\geq 0 \end{array}\right. \end{array}$$

where $L\left(y,f\left(X\right)\right)$ is the LaGrange function of a single out variable y as a function of n input variables X using a function $f\left(X\right)$. w is the weight parameter, b represents bias, and $\emptyset \left(X\right)$ is the transformation equation. C is a penalty factor that is used to control the accuracy of the regression model; if C is set to infinity, it means you are only concerned about accuracy rather than model complexity.

The SVR problem can be solved easily as a dual problem, and the kernel function $K\left(x_{i},x_{j}\right)$, which satisfies Mercer’s condition in the objective function, is used. Here, $\alpha_{i}$ and $\alpha_{i}^{*}\;$ are Lagrange multipliers, and data points with positive and non-zero $\alpha_{i}$ and $\alpha_{i}^{*}$ are support vectors.

In order to solve the optimization problem, the regression model is built as shown in Equation (7), where *b* is the bias of the SVR model.

(7) $$f(x)=\sum_{i=1}^{l}(\alpha_{i}^{*}-\alpha_{i})K(X,X_{i})+b$$

In Equation (7), the term $K\left(X,X_{i}\right)$ is known as the kernel function, and $X_{i}$ is the training sample, with X as an input variable. This kernel function should be chosen as a dot product in the high-dimensional feature space [67]. There are numerous types of kernel functions. The commonly used kernel functions for SVR are the linear kernel, polynomial kernel, radial basis function (RBF) kernel, and sigmoid kernel.

All of the kernel functions satisfy Mercer’s condition; however, the regression results of the kernels vary. Therefore, it is essential to choose the best kernel function for the SVR algorithm to obtain optimal performance.

### 3.5. KRR Model

KRR combines ridge regression with the kernel “trick”. This model can learn a linear function in the space induced by the respective kernel and the dataset. Nonlinear functions in the original space can be used by the nonlinear kernels. The KRR algorithm also analyzes several kernels such as the RBF kernel, sigmoid kernel, and polynomial kernel to find the suitable kernel function for the WLP nonlinear dataset.

The KRR is possibly the most elementary algorithm that can be kernelized to ridge regression [68]. In this study, a linear function that models the dependencies between the covariate input variable $x_{i}$and the response variable $y_{i}$ is found. The classic method is used to minimize the quadratic cost, as shown in Equation (8). However, for the nonlinear dataset, the lower-dimensional feature space replaces the higher-dimensional feature space; that is, $X_{i}\to \Phi \left(X_{i}\right)$. To convert lower-dimensional space to higher-dimensional space, the predictive model undergoes overfitting. Hence, to avoid overfitting, this function requires regularization.

(8) $$C(W)=\frac{1}{2}\sum_{i}{\left(y_{i}-W^{T}X_{i}\right)}^{2}$$

where $C\left(W\right)$ is the cost function of the weight-decay W and $W^{T}$ is the initial weight required for the input samples for the KRR model. A simple and effective way to regularize is to penalize the norm of W. This is called “weight-decay”, and it remains to be determined how to choose *λ* that is known as regularizing factor. Another way, the algorithm can be used cross-validation to avoid over-fitting. Hence, the total cost function becomes

(9) $$C=\frac{1}{2}\sum_{i}{\left(y_{i}-W^{T}X_{i}\right)}^{2}+\frac{1}{2}\lambda \left\|W^{2}\right\|$$

Equation (9) needs to be minimized. Therefore, the derivative of the equation must be obtained and then equated to zero.

To optimize the above cost function C, this study introduces Lagrange multipliers into the problem. Consequently, the derivation step becomes similar to that in the SVR case. After the optimization problem is solved, the resulting KRR regression model is shown in Equation (10).

(10) $$f(x)=\sum_{i=1}^{N}\alpha_{i}^{*}K(x,x^{i})$$

where $K\left(x,x^{i}\right)$ is the kernel function of the $x^{i}$ training sample with x as the input variable, and $\alpha_{i}^{*}$ is the weight of the KRR model and is equal to

(11) $$\alpha_{i}^{*}={\left(K+\lambda Ι\right)}^{-1}y$$

Hence, Equation (11) is very simple and more flexible due to introducing kernel function *K*, λ is the regularize factor with the identity matrix I, and y is the response variable. This model can also avoid both model complexity and computational time.

One big disadvantage of ridge regression is that there is no sparseness in the α vector; that is, there is no concept of support vectors. Sparseness is useful because when a new example is tested, only the support vectors need to be summed, which is much faster than summing the entire training set. In SVR, the only source of sparseness is the inequality constraints because, according to the complementary slackness conditions, if the constraint is inactive, then the multiplier $\alpha_{i}$ is zero.

### 3.6. KNN Model

The KNN model is a statistical tool for estimating the value of an unknown point based on its nearest neighbors [69]. The nearest neighbors are usually calculated as the points with the shortest distance to the unknown point [70]. Several techniques are used to measure the distance between the neighbors. Two simple techniques are used in this study: the Euclidean distance function $d\left(x,y\right)$, provided in Equation (12), and the Manhattan distance function $d\left(x,y\right)$, provided in Equation (13).

(12) $$d(x,y)=\left\|x-y\right\|=\sqrt{\sum_{i=1}^{n}{\left(x_{i}-y_{i}\right)}^{2}}$$

(13) $$d(x,y)=\sum_{i=1}^{n}\left|x_{i}-y_{i}\right|$$

where $x=\left(x_{1},\ldots ,x_{n}\right)$, $y=\left(y_{1},\ldots ,y_{n}\right)$, and n is the vector size. The *K* neighbor point that has the shortest distance to the unknown point is used to estimate its value using Equation (14).

(14) $${\hat{y}}_{i}=\sum_{i=1}^{n}w_{i}y_{i}$$

where $w_{i}$ is the weight of every single neighbor point $y_{i}$ to the query point $\hat{y}$ [71].

The KNN algorithm defined in Equation (14) is the weighted average of the neighborhood. The simplest KNN model is the mean of the contiguity, which is obtained in the case of uniform weights, where all of the neighbor points have the same effect on the estimation $w_{i}=\frac{1}{n}$. In contrast, when the neighbor points are assumed to have different effects on the query point estimation, different weights can be applied. The simplest weight function is provided in Equation (15).

(15) $$w_{i}=\frac{d_{i}}{\sum_{i=1}^{n}d_{i}}$$

where $d_{i}$ is the distance between the unknown point and its neighbor. The weight function must reach its maximum value at zero distance from the interpolated point, and as the distance increases, the function decreases [72].

The KNN estimation shown in Equation (14) depends only on the neighbor points; therefore, it neglects the trend of the whole dataset. However, Equation (15) provides better KNN estimations because the weighted distance considers a lower number of nearest values. Finally, this KNN algorithm is more suitable for regression and classification problems according to the simplest weight function.

### 3.7. The RF Regression Model

RF is a collection of decision trees. These tree models usually consist of fully grown and unpruned CARTs.

The structure of the RF regression model is shown in Figure 11. This algorithm creates an RF by combining several decision trees built from the training dataset. The CART tree selects one feature from all of the input features as the segmentation condition according to the minimum mean square error method.

**Figure 12:**
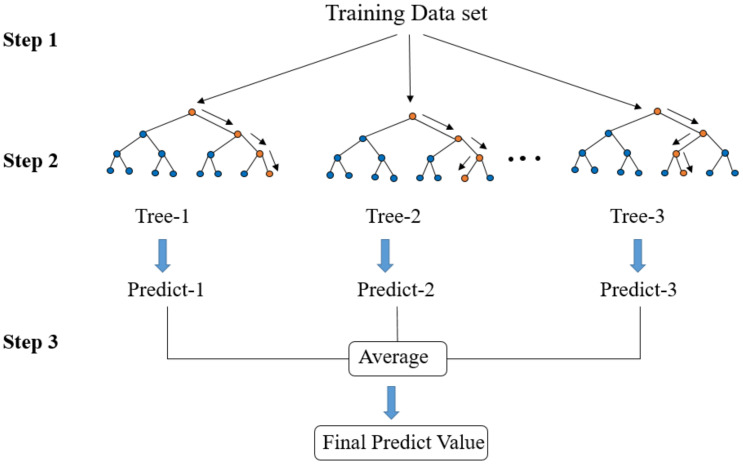
Schematic diagram of random forest structure.

The RF algorithm procedure comprises three steps. In step 1, the bagging method is used to create a subset that accounts for approximately 2/3 of the total data volume. In step 2, if the data value is greater than the selected feature value, the data points are separated to the right from the parent node, otherwise to the left of the parent node (Figure 12). Afterward, a set of trained decision trees is created. In step 3, the RF calculates the average value of all decision tree results to obtain the final predicted value.

### 3.8. Training Methodology

**Figure 13:**
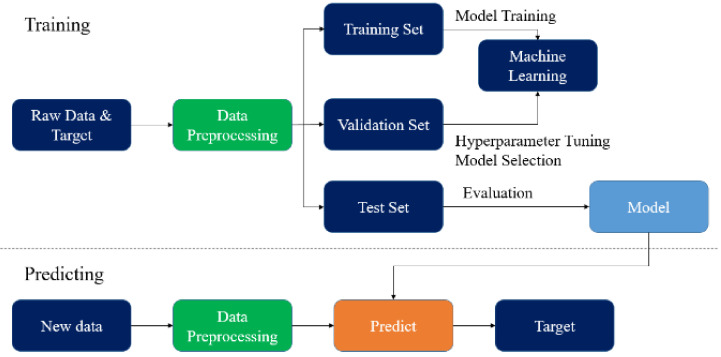
Methodology flow chart.

To obtain the best performance and avoid overfitting of the final trained regression model, several techniques, including data preprocessing (for standardization), cross-validation (for parameter selection), and grid search (for hyperparameter determination), were applied during training. The AI regression model was estimated using the method in the flowchart given in Figure 13.

#### 3.8.1. Data Preprocessing

The dataset values are not in a uniform range. Hence, before the machine learning model is developed, the data need to be preprocessed to standardize all of the input and output datasets and improve the modeling performance. Several data preprocessing methods, including min–max scaling, robust scaling, max absolute scaling, and standard scaling, were used in this study. Hence, of all preprocessing methods, we need to select the one method that provides the most accurately predicted output from the input dataset.

#### 3.8.2. Cross-Validation

**Figure 14:**
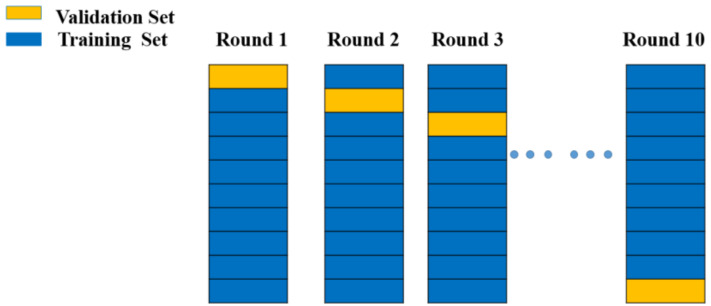
Cross-validation model diagram from Round 1 to Round 10.

Cross-validation is the most frequently used parameter selection method. The basic idea of cross-validation is that not all of the dataset is used for training; a part of it (which does not participate in training) is used to test the parameters generated by the training set. The training data are trained with different model parameters and verified by the validation set to determine the most appropriate model parameters. Cross-validation methods can be divided into three categories: the hold-out method, k-fold method, and leave-one-out method. Owing to the huge calculation burdens of the hold-out method and the leave-one-out method, the k-fold method was chosen for this study (Figure 14). After the choice of data preprocessing method was confirmed, cross-validation was performed to avoid overfitting of the machine learning model, as shown in Figure 14. The dataset was divided into 10 parts, and each part acted as either a validation or training set in different training steps. The validation sets were also used to predict the training results.

#### 3.8.3. Grid Search Technique

Grid search is a large-scale method for finding the best hyperparameter to build the training model. In order to determine the best parameter, the search range value needs to be set by the model builder. Although the method is simple and easy to perform, it is time-consuming. Therefore, to reduce the computation time, this work adopted the grid search technique to find the best hyperparameter as compared to manually searching the hypermeter, and eventually, the training model was fixed with the above hyperparameters to run the best AI model.

## 4. Results and Discussion

This section discusses the outcomes of the different machine learning algorithms used for the WLP structure design. In this study, we analyzed both the accuracy and CPU time consumption of the algorithms. Regarding accuracy, the mean absolute error (MAE) and the maximum absolute error between the FEM-predicted reliability life cycle and the AI-predicted reliability life cycle of the WLP structure were calculated in this work. We discuss both the training and testing error analysis for different datasets. Similarly, the CPU time required for every regression model to predict the WLP structure reliability life is also discussed. Training datasets comprising 576 and 1296 and a testing dataset comprising 54 WLP geometric combinations were used in this study.

Table 8 presents the ANN regression results for 576 training datasets. As shown in the table, different numbers of neurons, from 10 to 500, and hidden layers, from 2 to 20, were tested. After the numbers of hidden layers and neurons were tuned, the best ANN-predicted number of life cycles was calculated. Validated against the FEM results, the best MAE of AI prediction was five cycles, and the maximum absolute error was 28 cycles, with 10 hidden layers and 200 neurons. Similarly, Table 9 presents the ANN regression results for the training dataset with 1296. From the table, the best MAE was three cycles, and the maximum absolute error was 18 cycles, with five hidden layers and 500 neurons. Therefore, the ANN regression model based on the training dataset with 1296 numbers showed better accuracy than that based on the dataset with 576 numbers. Moreover, the CPU time performances for both datasets were different (Table 10). The CPU time required for 576 datasets was 151 s, whereas 235 s was required for 1296 datasets. Thus, although the dataset with a higher number of features resulted in higher accuracy, the CPU time was higher. 

Table 11 presents the results of the RNN regression models for the training dataset with 576 numbers. The RNN models considered different numbers of neurons and hidden layers. The best MAE was six cycles, and the maximum absolute error was 37 cycles, with 500 neurons and five hidden layers. Table 12 lists the RNN regression results for 1296 training datasets. From the table, the best MAE was three cycles, and the maximum absolute error was 27 cycles. Therefore, the RNN regression model based on the dataset with 1296 numbers exhibited better accuracy. However, the CPU time (698 s) was higher than that of the model based on 576 training datasets (173 s; Table 13). Given the above, the ANN regression model outperformed the RNN regression model in both accuracy and CPU time. This implies that the ANN model is more flexible than RNN because of the RNN model’s complex structure.

Table 14 presents the SVR results for 576 datasets. The table presents the accuracy and CPU time analysis results for the SVR model considering different kernel functions and different hyperparameters. The best MAE for the testing data was 13, and the maximum absolute error was 55 for the RBF kernel-based model. The shortest CPU time was 0.093 s. For the training dataset with 1296 numbers, the SVR exhibited better accuracy (Table 15). For this dataset, the best MAE for the testing data was 7.3 cycles, and the maximum absolute error was 30 cycles.

However, the larger dataset had a higher CPU time requirement. Given the above SVR results, we can infer that the RBF kernel plays a more important role in obtaining good SVR performance compared with the sigmoid and polynomial kernel functions.

Table 16 presents the KRR results for 576 training datasets. The table presents the accuracy and CPU time analysis for different kernel functions with hyperparameters used in the KRR algorithm. The best MAEs for the training and testing datasets were 8.4 and 12.2 cycles, respectively, for the model with the RBF kernel function. Moreover, the KRR model with the RBF kernel function required a short CPU time (0.093 s). The KRR model with the larger dataset showed better performance (Table 17). The best MAE of the test data was 5.6 cycles, and the maximum absolute error was 24 cycles for the training dataset with 1296. However, the CPU time was higher than that for the training dataset with 576 numbers (Table 18). Similar to the SVR results, the KRR results also show that the RBF kernel exhibited better accuracy and CPU time than the sigmoid and polynomial kernel functions. The KRR algorithm outperformed the SVR model in terms of accuracy and CPU time. Meanwhile, the ANN outperformed the RNN, SVR, and KRR in terms of accuracy. However, KRR and SVR outperformed ANN and RNN in terms of CPU time, owing to the usage of more hidden layers and a greater number of neurons are used in ANN and RNN. The KNN results are shown in Table 19. The table presents the accuracy of KNN in terms of the Euclidean and Manhattan distances versus the number of nearest neighbors (K) used in this algorithm for 576 training datasets. The best MAE and maximum absolute error were 18.2 and 72 cycles with the Manhattan distance when the K value was 9. From Table 19, the algorithm performs better in terms of the Manhattan distance than the Euclidean distance under the same K value. Similarly, Table 20 shows the results of KNN based on the dataset with 1296 numbers. From Table 20, the best MAE and maximum absolute error were 7.5 and 23 cycles, respectively, with the K value as 3. Table 21 compares the cases in which the weight as the uniform is used with the Euclidean distance and the weight as the distance is used with Euclidean distance. From the table, the case of the weight in terms of distance showed better MAE, and maximum absolute error than the case of the uniform weight is used for the KNN model of a WLP structure. With the increase in the number of training datasets from 576 to 1296, the model accuracy improved, whereas the CPU times remained similar, i.e., 0.03 and 0.034 s (Table 22). Table 23 compares the results of RF regression for the two training datasets. From the table, the MAE for the test data was 26.3 cycles for the 1296 dataset and 36 cycles for the 576 datasets. Therefore, the increase in the number of training datasets improved the accuracy. However, the CPU time was higher for the larger training dataset (Table 23).

**Figure 15:**
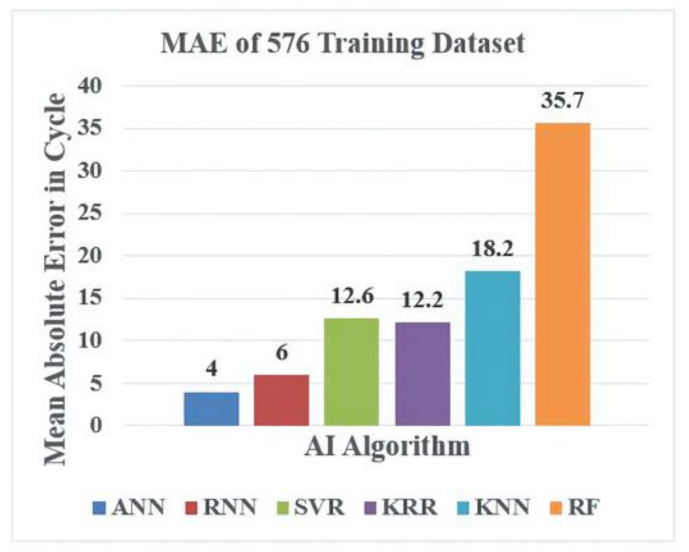
MAE analysis vs. AI model for 576 training datasets.

**Figure 16:**
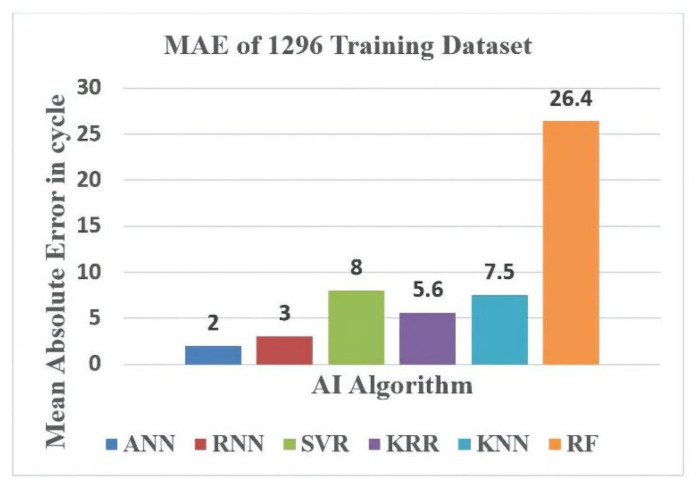
MAE analysis vs. AI model for 1296 training datasets.

Finally, error analysis and CPU time analysis were conducted for the different machine learning algorithms used to model the WLP structure. Figure 15 shows the MAE analysis with several AI algorithms for the 576-feature training dataset. From the figure, the ANN model exhibited the smallest error, i.e., 4, whereas the RF model exhibited the highest error, i.e., 35.7. The other AI model errors were close to that of the ANN model. Similarly, Figure 16 shows the MAE results for several AI algorithms with 1296 training datasets. From the Figure 16, the ANN model had the lowest MAE, i.e., 2, whereas the RF model had the highest error, i.e., 26.4, and the MAEs of the other AI models were close to that of the ANN model.

**Figure 17:**
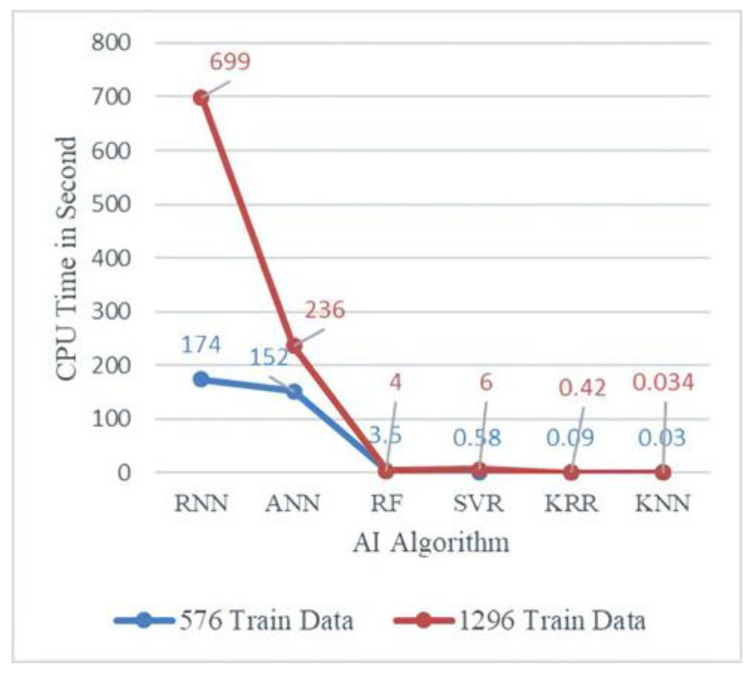
CPU time vs. AI algorithms for 576 and 1296 training datasets.

Furthermore, the CPU times required by the different AI algorithms for the WLP structure were investigated. Figure 17 presents the CPU time analysis results for different AI models based on the 576- and 1296-feature training datasets.

From Figure 17, for the 576 training dataset, the KNN model required the lowest CPU time (0.03 s), whereas the RNN model required the highest (174 s). The CPU times required by the RF, SVR, and KRR models were close to that of the KNN model. Similarly, for the 1296 training dataset, the KNN model required the least CPU time (0.034 s), whereas the RNN model required the highest CPU time (699 s). Again, the CPU times required by the RF, SVR, and KRR models were close to that of the KNN model. The ANN and RNN models required more CPU times than the KRR, SVR, KNN, and RF models because of the usage of more neurons and hidden layers to estimate the WLP lifetimes.

**Figure 18:**
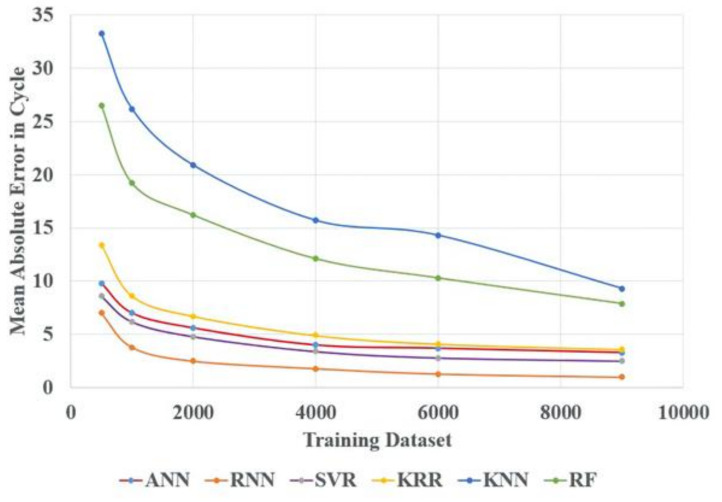
Mean absolute error vs. training dataset for different AI algorithms.

**Figure 19:**
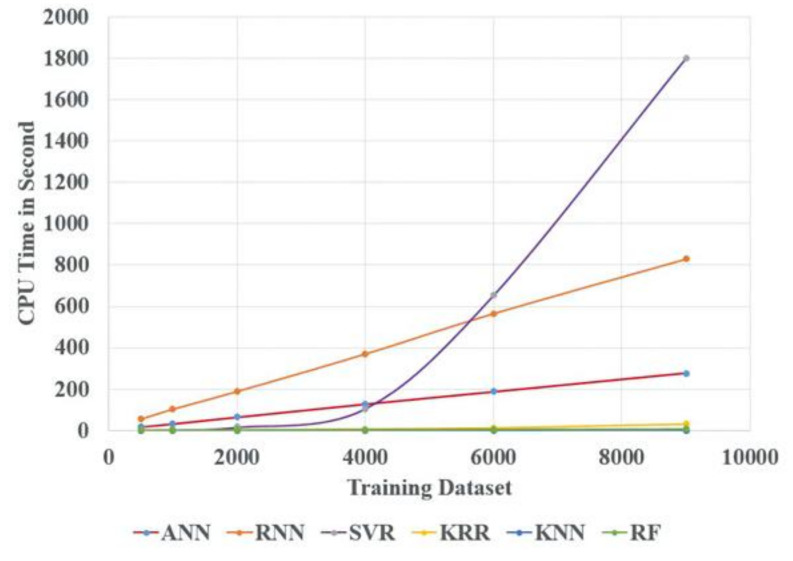
CPU time vs. training dataset for different AI algorithms.

Eventually, it also makes a comparison between all the above algorithms as per the MAE in cycle and CPU time consumption for different training datasets, which is from 500 to 9000 datasets. Figure 18 shows the MAE in cycle vs. different training datasets for all the above models. Figure 18 demonstrates the increase in the training dataset with the decrease in the testing error for all the above algorithms. From all the algorithms, ANN, RNN, SVR, and KRR testing errors are very close to each other. However, KNN and RF accuracy performance are not as good as other algorithms because these two algorithms are more suitable for classification purposes. Similarly, Figure 19 shows the CPU time analysis with different training datasets for all the above AI models. From Figure 19, it can be seen that CPU time increases with the increase in the training dataset. ANN and RNN required more CPU time due to the usage of more neurons and more hidden layers. The SVR algorithm also required more CPU time as compared to KRR, KNN, and RF algorithms due to the usage of more hyperparameters with the grid search technique to establish the best hyperparameter. 

Overall, the use of AI models may have a major impact on the electronic packaging industry. However, the result of the study confirms that a validated FEM solution procedure is crucial for generating reliable training datasets and that increasing the number of design features increases the CPU time needed to build the AI model for the WLP structure.

## 5. Conclusions

The machine learning algorithms used a large database generated by a validated FEM procedure for training analysis and obtained a structural reliability life cycle regression model for WLP. These AI regression models can predict the reliability life cycle of WLP structures when given the WLP geometry as the input. In terms of accuracy, the MAE between FEM and AI was less than 10 life cycles, which is acceptable, and the AI training CPU time consumption of several AI algorithms was small. The ANN algorithm exhibited the best accuracy, whereas the RF regression algorithm exhibited the lowest accuracy, presumably because RF is designed for classification purposes. Other algorithms such as KRR and SVR also showed good accuracy owing to the usage of the RBF kernel function. However, the KRR model slightly outperformed the SVR algorithm in terms of accuracy because of the use of fewer hyperparameters and lower model complexity. KNN is more suitable for classification purposes; nonetheless, the KNN regression model also exhibited good accuracy for the WLP structure database.

The study also investigated the CPU time required for training the above-mentioned AI algorithms to obtain a final regression model for predicting the reliability life of the newly designed WLP structure. The KNN, KRR, and RF regression models required less than 10 s, whereas the ANN, RNN, and SVR regression models required 150 to 1800 s. These times are very low compared with those required by FEM modeling and simulation, which can range from several hours to several days. 

It is known by the electronic packaging community that the experiment-based design procedure may take 8 months to a year to complete one run of the WLP test vehicle fabrication and the ATCT test; this has become unacceptable for todays’ advanced packaging development. Based on AI/machine learning algorithms and validated finite-element-based simulation technology, this research developed an AI-assisted design-on-simulation technology that can effectively and accurately predict the reliability life cycle of various geometries of WLPs. In addition, after obtaining the AI-trained model of the WLP, developers only need to input geometric data of the new WLP, then the reliability life cycle can be obtained within one second. Therefore, WLP structure optimization becomes feasible because the reliability prediction of any geometric combination of WLP can be completed in a few seconds. The AI-assisted design-on-simulation technology can also be applied to other packaging types such as system-in-packaging, heterogeneous, fan-out, WLP, and 3D packaging.

## Figures and Tables

**Table 1:** Dimension of WLP TV1 and TV2.

Material	TV1 (mm)	TV2 (mm)
Si Chip	5.3 × 5.3 × 0.33	4 × 4 × 0.33
Cu RDL	0.26 × 0.008	0.26 × 0.008
UBM	--	0.24 × 0.0086
Cu Pad	0.22 × 0.025	0.22 × 0.025
SBL1	5.3 × 5.3 × 0.0075	4 × 4 × 0.0075
SBL2	0.01	0.004
PCB	10.6 × 10.6 × 1	8 × 8 × 1
Low-k	5.3 × 0.005	4 × 0.005
Ball Diameter (mm)	0.25	0.25
Ball Pitch (mm)	0.4	0.4
Ball Counts	121	100
MTTF (cycles)	318	1013

**Table 2:** Dimension of WLP TV3, TV4 and TV5.

Material	TV3 (mm)	TV4 (mm)	TV5 (mm)
Si Chip	4 × 4 × 0.33	4 × 4 × 0.33	6 × 6 × 0.33
Cu RDL	0.18 × 0.004	0.2 × 0.004	0.25 × 0.0065
UBM	0.17 × 0.0086	0.19 × 0.0086	0.24 × 0.0075
Cu Pad	0.22 × 0.025	0.22 × 0.025	0.22 × 0.04
SBL1	4 × 4 × 0.0075	4 × 4 × 0.0075	6 × 6 × 0.008
SBL2	0.004	0.004	0.0065
PCB	8 × 8 × 1	8 × 8 × 1	12 × 12 × 1
Low-k	4 × 0.005	4 × 0.005	--
Ball Diameter (mm)	0.18	0.2	0.25
Ball Pitch (mm)	0.3	0.3	0.4
Ball Counts	144	144	196
MTTF (cycles)	587	876	904

**Table 3:** Material properties of WLP.

Material	Young’s Modulus (Gpa)	Poisson’s Ratio	CTE (ppm/°C)
Solder joint	Temperature-dependent	0.35	25
Silicon chip	150	0.28	2.62
Copper	68.9	0.34	16.7
Low-k	10	0.16	5
Solder mask	6.87	0.35	19
PCB	18.2	0.19	16

**Table 4:** Temperature-dependent Young’s modulus of SAC solder ball.

Temperature	Young’s Modulus (GPa)
233 K	45.74
253 K	42.22
313 K	31.66
353 K	24.62
398 K	16.70

**Table 5:** WLP finite element results for five test vehicles.

Test Vehicle	Experimental Reliability (Cycles)	Simulation Reliability (Cycles)	Difference
TV1	318	313	−5
TV2	1013	982	−31
TV3	587	587	0
TV4	876	804	72
TV5	904	885	19

**Table 6:** Training data 576 of FEM for four input features.

Feature Name	Level (mm)
Upper Pad Diameter	0.18, 0.20, 0.22, 0.24
Lower Pad Diameter	0.18, 0.20, 0.22, 0.24
Chip Thickness	0.20, 0.25, 0.30, 0.35, 0.40, 0.45
Stress Buffer Layer Thickness	0.0075, 0.0125, 0.0175, 0.0225, 0.0275, 0.0325
Total Number	576

**Table 7:** Training data 1296 of FEM for four input features.

Feature Name	Level (mm)
Upper Pad Diameter	0.18, 0.19, 0.20, 0.21, 0.22, 0.23
Lower Pad Diameter	0.18, 0.19, 0.20, 0.21 0.22, 0.23
Chip Thickness	0.20, 0.25, 0.30, 0.35, 0.40, 0.45
Stress Buffer Layer Thickness	0.0075, 0.0125, 0.0175, 0.0225, 0.0275, 0.0325
Total Number	1296

**Table 8:** Comparison results of FEM and ANN models on 576 training datasets.

	Hidden Layer (2)		Hidden Layer (5)		Hidden Layer (10)		Hidden Layer (20)	
Number of Neurons	Mean Absolute Error in Cycle	Maximum Absolute Error in Cycle	Mean Absolute Error in Cycle	Maximum Absolute Error in Cycle	Mean Absolute Error in Cycle	Maximum Absolute Error in Cycle	Mean Absolute Error in Cycle	Maximum Absolute Error in Cycle
10	57	148	62	157	65	182	51	138
20	60	164	65	179	14	69	10	60
50	63	159	11	51	8	54	11	67
100	66	173	7	51	5	31	9	40
200	64	173	7	47	5	28	5	40
500	10	62	5	43	7	35	7	46

**Table 9:** Comparison results of FEM and ANN models on 1296 training datasets.

	Hidden Layer (2)		Hidden Layer (5)		Hidden Layer (10)		Hidden Layer (20)	
Number of Neurons	Mean Absolute Error in Cycle	Maximum Absolute Error in Cycle	Mean Absolute Error in Cycle	Maximum Absolute Error in Cycle	Mean Absolute Error in Cycle	Maximum Absolute Error in Cycle	Mean Absolute Error in Cycle	Maximum Absolute Error in Cycle
10	51	142	47	137	47	125	14	57
20	47	140	47	143	12	50	9	57
50	47	127	22	35	7	42	5	43
100	23	104	6	35	4	24	5	27
200	13	70	4	31	3	22	4	27
500	6	43	3	18	3	20	3	19

**Table 10:** Comparison of ANN regression result for WLP.

Training Data Set (ANN)	Neuron Number	Hidden Layer	Mean Absolute Error in Cycle	Maximum Absolute Error in Cycle	CPU Time in Second
576	200	10	5	28	151
1296	500	5	3	18	235

**Table 11:** Comparison results of FEM and RNN models on 576 training datasets.

	Hidden Layer (2)		Hidden Layer (5)		Hidden Layer (10)		Hidden Layer (20)	
Number of Neuron	Mean Absolute Error in Cycle	Maximum Absolute Error in Cycle	Mean Absolute Error in Cycle	Maximum Absolute Error in Cycle	Mean Absolute Error in Cycle	Maximum Absolute Error in Cycle	Mean Absolute Error in Cycle	Maximum Absolute Error in Cycle
10	65	166	61	178	61	159	26	101
20	63	163	62	168	13	54	16	64
50	65	157	29	71	8	57	10	43
100	65	159	12	71	7	37	8	65
200	17	79	8	50	9	53	10	65
500	12	69	6	36	6	37	9	50

**Table 12:** Comparison results of FEM and RNN models on 1296 training datasets.

	Hidden Layer (2)		Hidden Layer (5)		Hidden Layer (10)		Hidden Layer (20)	
Number of Neuron	Mean Absolute Error in Cycle	Maximum Absolute Error in Cycle	Mean Absolute Error in Cycle	Maximum Absolute Error in Cycle	Mean Absolute Error in Cycle	Maximum Absolute Error in Cycle	Mean Absolute Error in Cycle	Maximum Absolute Error in Cycle
10	125	355	44	141	11	61	6	44
20	69	215	21	103	10	54	10	57
50	33	154	5	28	4	30	4	30
100	10	51	3	28	4	32	4	32
200	4	36	4	32	6	43	4	32
500	3	27	3	39	159	506	159	506

**Table 13:** Comparison of RNN regression results for WLP.

Training Data Set (RNN)	Neuron Number	Hidden Layer	Mean Absolute Error in Cycle	Maximum Absolute Error in Cycle	CPU Time in Second
576	500	5	6	36	173
1296	500	2	3	27	698

**Table 14:** Comparison results of FEM and SVR models on 576 training datasets.

SVR Kernel Function	RBF Kernel	Sigmoid Kernel	Polynomial Kernel Degree 3
Hyperparameter (C)	2250.97	540.31	2563
Hyperparameter (γ)	0.86	1.7	4
Hyperparameter (ε)	10	10	10
Training Score ($R^{2}$)	0.996	0.97	0.976
Cross-Validation Score ($R^{2}$)	0.964	0.936	0.954
Maximum Absolute Error (cycle) Train Data	68	125	142
Mean Absolute Error (cycle) Train Data	10	30	25
Maximum Absolute Error (cycle) Test Data	55	93	94
Mean Absolute Error (cycle) Test Data	13	29	20
CPU Time In Second	0.093	0.119	0.153

**Table 15:** SVR result comparison.

SVR Training Dataset	576	1296
Hyperparameter (C)	2250.97	3000.51
Hyperparameter (γ)	0.86	2.37
Hyperparameter (ε)	10	10
Training Score ($R^{2}$)	0.996	0.998
Cross-Validation Score ($R^{2}$)	0.964	0.981
Maximum Absolute Error (cycle) Train Data	68	46
Mean Absolute Error (cycle) Train Data	10	7.3
Maximum Absolute Error (cycle) Test Data	55	30
Mean Absolute Error (cycle) Test Data	13	8
CPU Time In Second	0.093	6.00

**Table 16:** Comparison results of FEM and KRR models on 576 training datasets.

KRR Kernel Function	RBF Kernel	Sigmoid Kernel	Polynomial Kernel Degree 3
Hyperparameter (α)	0.01	8.16	0.1
Hyperparameter (γ)	1	0.09	3.9
Maximum Absolute Error (Cycle) Train Data	57	149	75
Mean Absolute Error (Cycle) Train Data	8.4	38.7	18.9
Maximum Absolute Error (Cycle) Test Data	39	84	57
Mean Absolute Error (Cycle) Test Data	12.2	25.4	17.9
CPU Time In Second	0.093	0.117	0.157

**Table 17:** Comparison results of FEM and KRR models on 1296 training datasets.

KRR Kernel Function	RBF Kernel	Sigmoid Kernel	Polynomial Kernel Degree 3
Hyperparameter (α)	1 $\times e^{-10}$	3 $\times e^{-9}$	1 $\times e^{-9}$
Hyperparameter (γ)	0.19	0.02	2
Maximum Absolute Error (Cycle) Train Data	40	46	107
Mean Absolute Error (Cycle) Train Data	5.3	7.2	16
Maximum Absolute Error (Cycle) Test Data	24	29	45
Mean Absolute Error (Cycle) Test Data	5.6	7	14.5
CPU Time In Second	0.422	1.495	0.787

**Table 18:** KRR result comparison.

KRR Training Dataset	576	1296
Hyperparameter (α)	0.01	1 $\times e^{-10}$
Hyperparameter (γ)	1	0.19
Maximum Absolute Error (Cycle) Train Data	57	40
Mean Absolute Error (Cycle) Train Data	8.4	5.3
Maximum Absolute Error (Cycle) Test Data	39	24
Mean Absolute Error (Cycle) Test Data	12.2	5.6
CPU Time In Second	0.093	0.422

**Table 19:** Comparison results of FEM and KNN models on 576 training datasets.

Number Nearest Neighbor (K)	Euclidean Distance		Manhattan Distance	
	Mean Absolute Error in Cycle	Maximum Absolute Error in Cycle	Mean Absolute Error in Cycle	Maximum Absolute Error in Cycle
1	53.7	172	63	172
2	59.3	128	56.9	124
3	38.2	115	37.9	83
4	28.9	81	28.1	81
5	25.5	74	24.2	66
6	27.6	71	25.6	67
7	22.3	71	21.4	63
8	21.6	77	20.5	77
9	18.9	83	18.2	72
10	21.4	81	18.7	77

**Table 20:** Comparison results of FEM and KNN models on 1296 training datasets.

Number Nearest Neighbor (K)	Euclidean Distance		Manhattan Distance	
	Mean Absolute Error in Cycle	Maximum Absolute Error in Cycle	Mean Absolute Error in Cycle	Maximum Absolute Error in Cycle
1	2626.37	172	26.3	74
2	18.2	55	14.3	51
3	13	46	7.55	23
4	20.2	54	11.62	41
5	17.8	43	11.15	51
6	14.5	44	11.86	44
7	12.7	37	13.87	52
8	11.5	38	13.78	42
9	11.4	28	11.93	41
10	13.7	36	11.02	42

**Table 21:** KNN result comparison with different weights.

Number Nearest Neighbor (K)	Weight as Uniform with Euclidean		Weight as Distance with Euclidean	
	Mean Absolute Error in Cycle	Maximum Absolute Error in Cycle	Mean Absolute Error in Cycle	Maximum Absolute Error in Cycle
9	2618.9	8374	18.2	72

**Table 22:** KNN result comparison with different datasets.

Training Dataset(KNN)	Nearest Neighbor Value (K)	Mean Absolute Error in Cycle	Maximum Absolute Error in Cycle	CPU Time in Second
576	9	18.2	72	0.03
1296	3	7.5	23	0.034

**Table 23:** RF result comparison with different datasets.

Random Forest Training Dataset	576	1296
Random State	1	1
Number of Tree	81	81
Maximum Absolute Error (Cycle) Train Data	56	28
Mean Absolute Error (Cycle) Train Data	12	6.3
Maximum Absolute Error (Cycle) Test Data	133	103
Mean Absolute Error (Cycle) Test Data	36	26.3
CPU Time In Second	3.5	4
